# Neutrophil activation and circulating neutrophil extracellular traps are increased in venous thromboembolism patients for at least one year after the clinical event

**DOI:** 10.1007/s11239-021-02526-z

**Published:** 2021-08-27

**Authors:** Kiara C. S. Zapponi, Fernanda A. Orsi, José Luiz R. Cunha, Ingrid R. de Brito, Anna Virginia C. Romano, Luis Fernando Bittar, Erich Vinicius De Paula, Carla F. Penteado, Silmara Montalvão, Joyce Maria Annichino-Bizzacchi

**Affiliations:** 1 Hematology and Hemotherapy Center, University of Campinas, Carlos Chagas street, 480, Campinas, 13083­878 Brazil; 2 Department of Clinical Pathology, School of Medical Sciences, University of Campinas, Campinas, SP Brazil; 3 Leiden University Medical Center (LUMC), Leiden, The Netherlands

**Keywords:** Venous thromboembolism, Neutrophil, Adhesion, Chemotaxis, Reactive oxygen species, Extracellular traps, Endothelial dysfunction

## Abstract

Neutrophil activation and neutrophil extracellular traps (NETs) have been associated with the pathogenesis of venous thromboembolism (VTE). Considering VTE-associated chronic sequelae, which suggest that some pathological mechanisms remain after the acute episode, we investigated whether neutrophil activation is increased in patients with a prior VTE at least one year before this investigation. Thirty-seven patients with prior VTE and 37 individuals with no history of VTE were included. Neutrophil activity was evaluated by the expression of the adhesive molecule activation-specific epitopes LFA-1 (CD11a) and MAC-1 (CD11b), chemotaxis, reactive oxygen species (ROS) and by MPO-DNA complexes as markers of NETs. The adhesive molecules sICAM-1 and sVCAM-1, involved in the cross talk between neutrophil and endothelial cells, were also evaluated. Patient neutrophils presented increased CD11a expression before and after TNF-α stimulus, whereas increased CD11b expression was observed only after TNF-α stimulus, as compared to controls. Neutrophil chemotaxis on both, basal state and after IL-8 stimulus, on circulating levels of sICAM-1 and sVCAM-1, and on MPO-DNA complexes were also increased in VTE patients. ROS release was similar between patients and controls. This is, to our knowledge, the first study to investigate neutrophil inflammatory activity in VTE patients a long period after an acute event (approximately 2 years). The results showed altered neutrophil activation patterns in these patients. While activated neutrophils can cause endothelial activation and injury, the activated endothelium can induce the release of NETs with consequent endothelial cytotoxicity, creating a vicious cycle of activation between neutrophils and endothelium that can lead to thrombosis.

**Graphical abstract:**

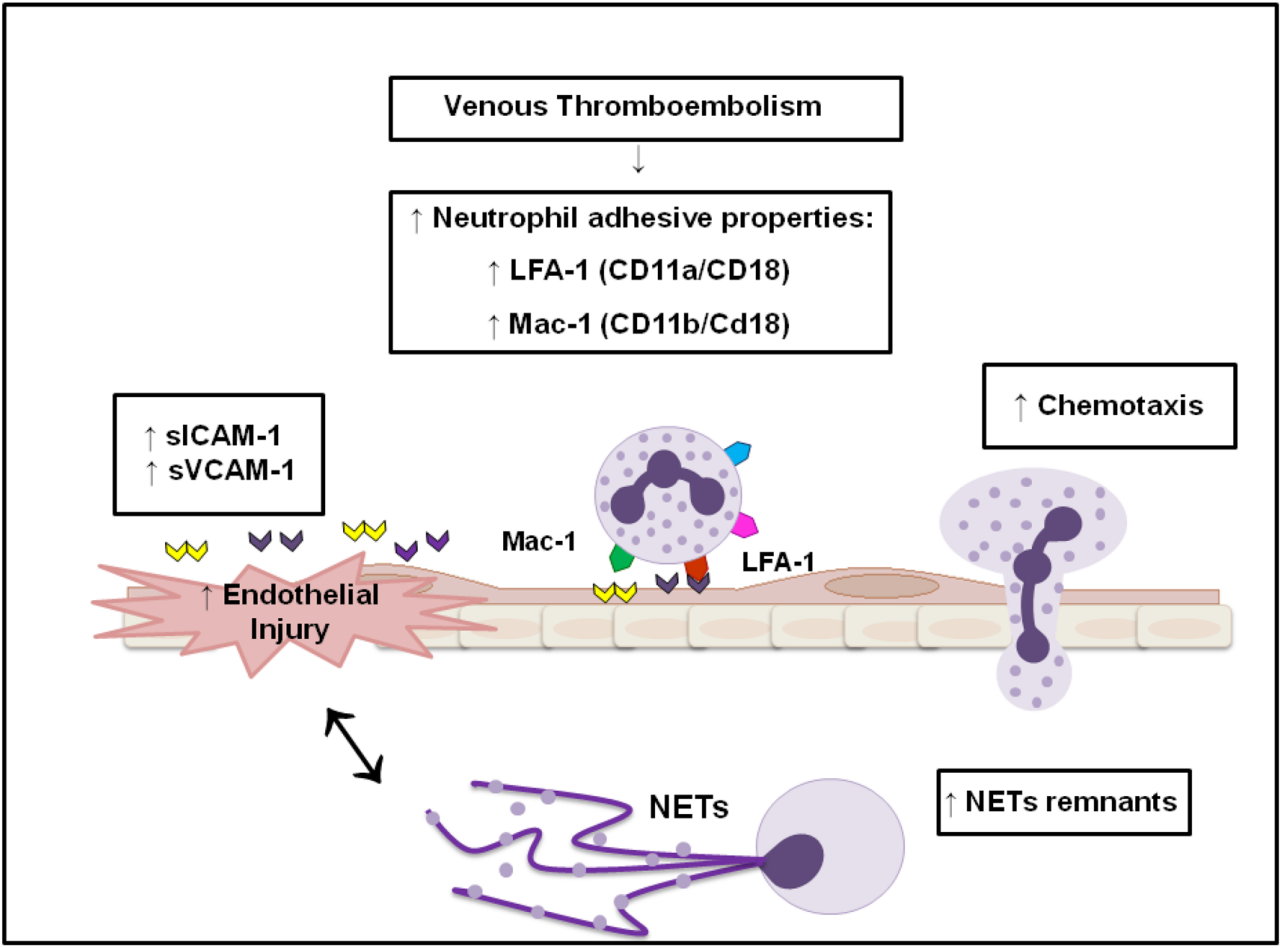

VTE patients (approximately 2 years after the clinical event) present an altered neutrophil activation state evidenced by increased activity of the LFA-1 and Mac-1 adhesive molecules, as well as increased chemotaxis and circulating levels of NETs remnants. Circulating levels of ICAM-1 and VCAM-1, which are endothelial adhesive molecules, are also increased in VTE patients, suggesting not only an exacerbated endothelial activation and dysfunction, but also an interaction of the neutrophil adhesive molecules with their endothelial ligands, favoring the migration process of neutrophil.

## Highlights

- Increased neutrophil adhesive and chemotactic properties were observed in patients 2 years after the acute VTE event.
- Increased levels of the MPO-DNA complex (NETs remnants marker) were found in the serum of patients with a prior VTE (≅ 2 years).
- VTE patients showed increased levels of sICAM-1 and sVCAM-1, molecules associated with endothelial activation.

## Introduction

Venous thromboembolism (VTE) is associated with long-term clinical complications, such as post-thrombotic syndrome (PTS) [1, 2] and recurrent VTE [3, 4], with pathological mechanisms that are yet to be fully elucidated [3].

The neutrophils are reported to be the first cells recruited to the site of inflammation, and many studies have demonstrated their substantial role in the thrombus formation [5]. Neutrophils are quiescent in nature and their activation can be considered a two-step process, in which exposure to a stimulus (priming) guarantees a maximum response to one-second stimulus [6]. The first activation pathway is initiated by neutrophil adhesion to the endothelium, followed by firm attachment and migration to extravascular tissues. The firm adherence is mediated by interactions of β2 integrin expressed on the neutrophil plasma membrane, mainly by the subunits LFA-1 (CD11a/CD18) and Mac-1 (CD11b/CD18), with the intercellular adhesion molecule-1 (ICAM-1) and vascular cell adhesion protein-1 (VCAM-1) on the activated endothelium [7, 8].

After adhesion, a second pathway of activation includes neutrophil effectors functions, such as generation of reactive oxygen species (ROS), degranulation, phagocytosis and release of large amounts of chemokines. In addition to phagocytosis, neutrophils are also capable of responding to inflammation through release of neutrophil extracellular traps (NETs) [9]. Following an inflammatory stimulus, the generation of ROS can lead to the dissolution of intracellular membranes and translocation of myeloperoxidase (MPO) and elastase to nucleus. Histones are then hyper-citrullinated by the peptidyl arginine deiminase 4 (PADI4) enzyme and degraded by elastase, promoting chromatin decondensation, plasma membrane rupture and NETs release. Therefore, NETs consist of antimicrobial structures of DNA lined with granular components [10].

NETs are further alleged to play a role in coagulation [11]. Activation and excessive formation of NETs have been associated with endothelial injury [12–14] and thrombotic disorders [15–18]. Previous studies demonstrated that activated neutrophils and NETs participate in thrombus development by inferior vena cava (IVC) ligation in mice [19]. Clinical studies have further demonstrated that circulating levels of NETs are increased during VTE acute episodes [20–22]. Considering these previous findings, we evaluated whether neutrophil activity and NETs are detected in patients long-term after the VTE acute episode.

## Methods

### Study participants

From March 2014 to February 2015, patients with a prior diagnosis of VTE assisted at the Hematology and Hemotherapy Center of University of Campinas (UNICAMP) were selected for the study. Exclusion criteria included individuals under the age of 18 or above 70 years at the time of VTE, thrombosis at unusual sites, cancer, severe liver or kidney disease, autoimmune diseases such as antiphospholipid syndrome and pregnancy. Clinical and laboratory parameters, such as hereditary thrombophilia, site of thrombosis (deep vein thrombosis [DVT] or pulmonary embolism [PE]), and comorbidities were retrieved from the medical records. Participants with no prior thrombosis (controls) were selected among employees, volunteers and blood donors at UNICAMP. We matched patients and controls not only by age and sex, but also by the presence of cardiovascular risk factors, as hypertension and dyslipidemia. Exclusion criteria were the same as for patients. All eligible subjects were enrolled for the study after providing written informed consent, approved by the ethics committee of UNICAMP.

### Sample collection

Pre-analytical procedures were performed to mitigate any problem with the samples collection and handling. In order to avoid possible interfering influences samples from patients and their respective controls were collected on the same day and immediately processed after venipuncture (within 1 h) under the same conditions.

The blood collection was performed with the butterfly vein cannula (21G; 450,081, Greiner Bio-One, Austria). To evaluated plasma D-dimer and C Reactive Protein (hs-CRP) and sICAM-1/sVCAM-1 levels in the serum, blood samples were collected into Vacuette tubes (Greiner Bio-One, Austria): 0.129 mmol/L trisodium citrate tube and Z Serum Sep Clot Activator tube, respectively. The samples were centrifuged for 15 min at 1500 g, plasma and serum were stored at − 80 °C.

To evaluate the MPO-DNA complex, the aliquots of serum were treated with 10 mM EDTA (pH 8) immediately after centrifugation to inhibit further fragmentation of the DNA (good stability) [23].

We used 3 heparin-containing vacutainer tubes (10 ml; 14.4 USP units/ml of blood; BD Biosciences, New Jersey, USA), that are indicated when working with neutrophil activity assays [24], to evaluated adhesive and chemotactic properties of neutrophil as well as ROS assay.

### Neutrophil isolation

Neutrophil isolation, peripheral blood samples (6 ml) were immediately placed over two layers of ficoll-paque of density of 1.077 (3 ml) and 1.119 g/L (3 ml) (Sigma-Aldrich). After separation of monocytes and granulocytes by centrifugation at 700 g for 30 min, at room temperature, contaminating erythrocytes were lysed (10 min, 4 °C, lysis buffer: 155 mM NH_4_Cl, 10 mM KHCO_3_) and cells were then washed in phosphate-buffered saline (PBS; pH 7.4) and resuspensed in RPMI 1640 medium (1640 Vitrocell Embriolife) [25]. Cells were counted using the Advia Hematology System (Bayer, Tarrytown, NY, USA). Purity analysis of isolated neutrophil was evaluated after cytospin process (300 RPM, 5 min, Shandon Cytospin 4, Thermo). The viability > 98% was obtained by counts of viable and non-viable cells stained with methylene blue using the Neubauer chamber. Neutrophil suspensions were utilized immediately in the neutrophil activation assays only when their purity was greater than 92%.

### Laboratory Procedures

D-dimer plasma levels were determined by immunoturbidimetric analysis, as recommended by the manufacturer, in an automated coagulation analyzer (BCS XP, Siemens, Marburg, Germany). Normal laboratory values were considered levels ≤ 550 ng/ml.

The hs-CRP levels were determined by a nephelometric method (Siemens, Marburg, Germany), on Siemens BN ProSpec analyzer.

### Analysis of neutrophil activation

#### Neutrophil adhesive properties by flow cytometry

Isolated neutrophils (1 × 10^6^ cells/ml) were co-incubated or not with recombinant human TNF-α (R&D Systems, 210-TA-020) (200 ng/ml, 30 min, 37 °C, 5% CO_2_). To detect molecules in their activated conformations by activation specific epitopes, CD11a and CD11b, the cells were incubated with either APC-conjugated mouse anti-human CD11b antibody (17–0113-42, eBioscience) [26] or mouse anti-CD11a (ab3981, ABCAM)/FITC-conjugated anti-mouse IgG1 (ab11588, ABCAM) for 30 min at 4 °C, in the dark [27]. The cells were read in a FACScalibur cytometer (BD Biosciences, EUA) at 488 nm and were analyzed using Flowjo Software. Gating strategies SSC/FSC (side scatter/forward scatter), dot plots were used to identify the neutrophil population. Data are expressed as mean fluorescence intensities (MFI) compared to a negative isotype control.

#### In vitro neutrophils chemotaxis

For the cell migration assays in vitro 96-well chemotaxis chambers were used (Chemo Tx; Neuro probe, Gaithersburg, MD, USA). Twenty nine microliters of chemotactic agent IL-8 (618-IL-050 Recombinant Human CXCL8/IL-8, R&D Systems) (100 ng/ml) and RPMI 1640 medium (control for spontaneous migration) were pipetted to the bottom of the plate and a polycarbonate filter (5 µm pore) placed over the wells. Twenty-five microliters of neutrophil suspension in RPMI medium (4 × 10^6^ cell/ml contain no FBS/FCS) were then added to the chamber’s upper compartment. Chambers were incubated for 120 min (37 °C, 5% CO_2_) and the wells of the upper compartment containing the remaining cells, which did not migrate to the lower, were emptied by aspiration. To detach adherent neutrophils from the lower surface of the filter, the microtiter plate with the attached filter was centrifuged for 5 min (290 g, at room temperature). The contents of the wells were resuspended in RPMI to a final volume of 29 μl and transferred to a flat-bottomed ELISA plate. A standard curve of known cell concentrations was also added, where the concentration of 2 × 10^6^ cel/ml was equivalent to the highest point of the curve (100%). The MPO extraction from neutrophil was carried out by adding 0.5% HTAB (14,5 μl -Hexadecyltrimethylammonium bromide) to 50 mM phosphate buffer pH 6.0 and plates were then stored frozen overnight. After being thawed, the number of migrated cells was estimated by measuring the MPO content by assayed spectrophotometrically: 10 μl of the material to be measwed was mixed with 190 μl of 50 mM phosphate buffer, pH 6.0, containing 8,35 mg/ml o-dianisidine dihydTochloride (Sigma Chemical Co.) and 0.0005% hydrogen peroxide, as described elsewhere [28]. Migrated neutrophils were calculated by comparing absorbance 492 nm (Versamax; Molecular Devices, Sunnyvale, CA, USA) of unknown samples with those of the standard curve, determined by the MPO values measurements. The results were expressed as a percentage (%) of migrated neutrophil.

#### MPO-DNA complexes

MPO-DNA complexes were detected by capture ELISA [29]. Anti-MPO antibody (5 µg/ml, ABD Serotec, Cat-No. 0400-0002) was coated to 96-well microtiter plates overnight at 4 °C. After blocking with 1% BSA in PBS (30 min, 37 °C), patient’s sera was added to each well at a 1:20 dilution in peroxidase-labelled anti-DNA monoclonal antibody (component n° 2 of the cell death detection ELISA kit, Roche, Cat. No: 11774425001). The plate was incubated for 2 h at room temperature using a shaking device (320 rpm). The samples were subsequently washed three times with PBS and the peroxidase substrate (ABTS) contained in the kit (Roche, Cat. No: 11774425001) was added. After 40 min’ incubation at 37 °C in the dark, the absorbance was measured at 405 nm wavelength using VersaMax™ Microplate Reader (Molecular Devices, USA).

#### Measurement of neutrophil intracellular reactive oxygen species formation

The 2′-7′-Dichlorodihydrofluorescein diacetate (H2DCFH-DA; Invitrogen Corp., Carlsbad, CA, USA) kit was used to directly measure ROS in neutrophils. After cleavage of the acetate groups by intracellular esterases and oxidation, the non-fluorescent H2DCFDA is converted to highly fluorescent 2′, 7′-dichlorofluorescein (DCF). Neutrophils (1 × 10^6^ cells/ml) were incubated with 100 µM (final concentration) H2DCFH-DA for 15 min at 37 °C, 5% CO^2^. Cells were centrifuged (400 g, 5 min), resuspended in PBS and the formation of ROS was immediately analyzed by flow cytometry (FACSCalibur cytometer) using the FL1 channel (green fluorescence). Gating strategies SSC/FSC (side scatter/forward scatter), dot plots were used to identify the neutrophil population. The results were presented by MFI. Gating strategies SSC/FSC (side scatter/forward scatter), dot plots were used to identify the neutrophil population. The results were presented by MFI.

### Quantification of sICAM-1 and sVCAM-1 endothelial adhesive glycoproteins

Soluble ICAM-1 and VCAM-1 (sICAM-1 and sVCAM-1) were quantified in the patients’ serum using the Human Cardiovascular Disease (CVD) Panel 2 Magnetic Bead Kit (MILLIPLEX MAP kit, cat #HCVD2MAG-67K, Millipore, EUA). Plate reading was performed on the Luminex 200 equipment (Luminex Corp., USA) and results were presented as MFI.

### Statistical analysis

Statistical analyses were performed using GraphPad Prism 5.00.288 program (GraphPad Software, Inc, La Jolla, CA). To approach whether the data was normally distributed, we used the Shapiro–Wilk test, p value > 0.05 indicated data normally distribution. We selected the use of parametric tests only when both groups (patients × controls) presented data with normally distribution, otherwise non-parametric tests were used. Differences between groups were evaluated by Mann–Whitney U or T student and Fisher’s exact test. The Wilcoxon matched pairs test was used to compare groups before and after treatment with specific drugs. In descriptive statistics, all reported results are in median and interquartile range (IQR 25th–75th). Statistical significance was established as p ≤ 0.05.

## Results

### Demographic, clinical and laboratorial characteristics

**Fig. 1:**
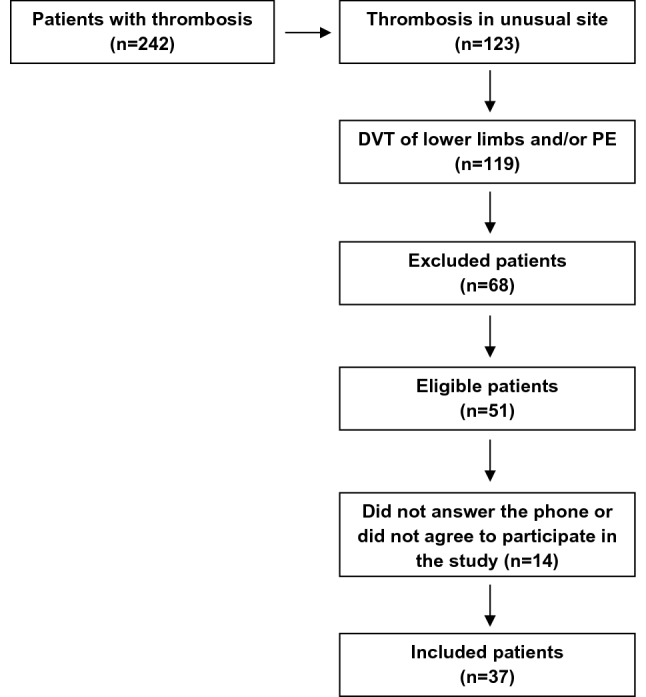
Fluxogram of VTE patients inclusion in the study

A total of 242 patients were evaluated for inclusion. Among the 119 patients with a prior VTE event, 51 met the inclusion criteria and 37 patients agreed to participate in the study (Fig. 1). Additionally, 37 controls matched by gender and age were selected. The median age among patients was 43 years and 13 (35.13%) were male. Demographic parameters were similar between patients and controls. The median time elapsed from VTE event to study inclusion was 24 months. Eighteen patients (48%) presented spontaneous VTE. DVT was observed in 28 patients (75.65%), and 9 patients (24.32%) had PE alone. Hereditary thrombophilia was detected in 8 (21.62%) patients: 5 heterozygous Factor V Leiden, 2 heterozygous F2 20210A and one protein S (PS) deficiency. The prevalence of comorbidities such as dyslipidemia and arterial hypertension, as well as smoking habits and alcohol use, was similar between patients and controls. As expected, a higher D-dimer was observed in VTE patients compared to controls (p < 0.0001), with 48.44% of patients with levels above 500 ng/ml versus 8% of controls. C-reactive protein (CRP), an acute inflammatory protein, was also higher in these patients when compared to controls (p < 0.0001). White blood cell (WBC) counts were higher in VTE patients 6.64 × 10^3^ cell/μl (IQR 6.02–8.03) than in controls 5.67 × 10^3^ cell/μl (IQR 4.75–6.48) (p = 0.005). However, these values are within the normal range (female: 3.9–11.1 × 10^3^/μl/male: 3.7–9.5 × 10^3^/μl) (Table 1).

**Table 1:** Laboratory and clinical characteristics of study subjects

	Controls (n = 37)	VTE patients (n = 37)	p
Demographic data, n			
Age median (years)^**a**^	44 (21–66)	43 (19–65)	0.94
Male/female	13/24	13/24	1
Caucasian/non-Caucasian	29/8	29/8	1
Comorbidities, n (%)	14 (37.83)	19 (51.35)	0.35
Dyslipidemia/hypertension	7 (18.91)	17 (45.94)	
Hypotireoidism	2 (5.4)	3 (8.10)	
Diabetes	0 (0)	5 (13.51)	
Others	4 (10.81)	3 (8.10)	
Thrombosis, n (%)			
Spontaneous VTE		18 (48.64)	
Median time after VTE^**a**^		24 (13–42)	
DVT		28 (75.65)	
PE		9 (24.32)	
Anticoagulation therapy	0	9 (24.32)	
Heparin		3 (33.33)	
Rivaroxaban		4 (44.44)	
Warfarin		2 (22.22)	
Laboratory parameters			
D-dimer (ng/ml)	250 (170–337)	493.2 (330.8–775)	< 0.0001
D-dimer (> 550 ng/ml)	3 (8.10)	18 (48.64)	
C-reactive protein (CRP-mg/L)	1.1 (0.2–2.27)	4.2 (1.3–9.7)	< 0.0001
WBC (F: 3.9–11,1/M: 3.7–9.5 × 10^3^ cell/μl)	5.67 (4.75–6.48)	6.64 (6.02–8.03)	0.005
Medication used	19 (51.35)	27 (72.97)	0.055
Levothyroxine/thiamazole	1/1 (10.52)	3/0 (11.11)	
Antihypertensive drugs	2 (10.52)	15 (55.55)	
NSAID	1 (5.26)	2 (7.40)	
Gastric protection	1 (5.26)	6 (22.22)	
Statins	1 (5.26)	5 (18.51)	
Insulin/metformin	0	2/3 (18.51)	
Psychotropics	2 (10.52)	5 (18.51)	

Continuous variables are displayed as median and interquartile range (25th–75th). Categorical variables are displayed as counts and percentages (%)

*DVT* deep vein thrombosis, *PE* pulmonary embolism, *VTE* venous thromboembolism, *WBC* white blood cells, *F* female, *M* Male, *NSAID* non-steroidal anti-inflammatory drugs

^a^Results presented as median (min–max). The p value were calculated by Mann–Whitney and Fisher’s exact test

In addition, only 32 of these 37 patients had enough cell numbers (isolated neutrophils) for neutrophil adhesion, chemotaxis, and ROS assays, as well as 32 serum samples available for endothelial activation test.

### Neutrophil activation

#### Increased adhesive and chemotactic properties of neutrophil in VTE patients

After inflammatory stimulation with TNF-α, expression of CD11a and CD11b increased in both VTE patients and controls. In VTE patients, the expression of CD11a increased from 33.70 (IQR 27.83–39.45) to 37.45 (IQR 33.43–43.60; p = 0.006) and the expression of CD11b increased from 108.00 (IQR 69.43–138.50) to 178.00 (IQR 141.80–248.50; p < 0.001). whereas in controls, the expression of CD11a increased from 30.30 (IQR 26.05–33.90) to 30.90 (IQR 28.43–39.00; p = 0.05) and the expression of CD11b increased from 99.20 (IQR 71.10–120.00) to 147.50 (IQR 108.90–178.30; p < 0.001).

**Fig. 2:**
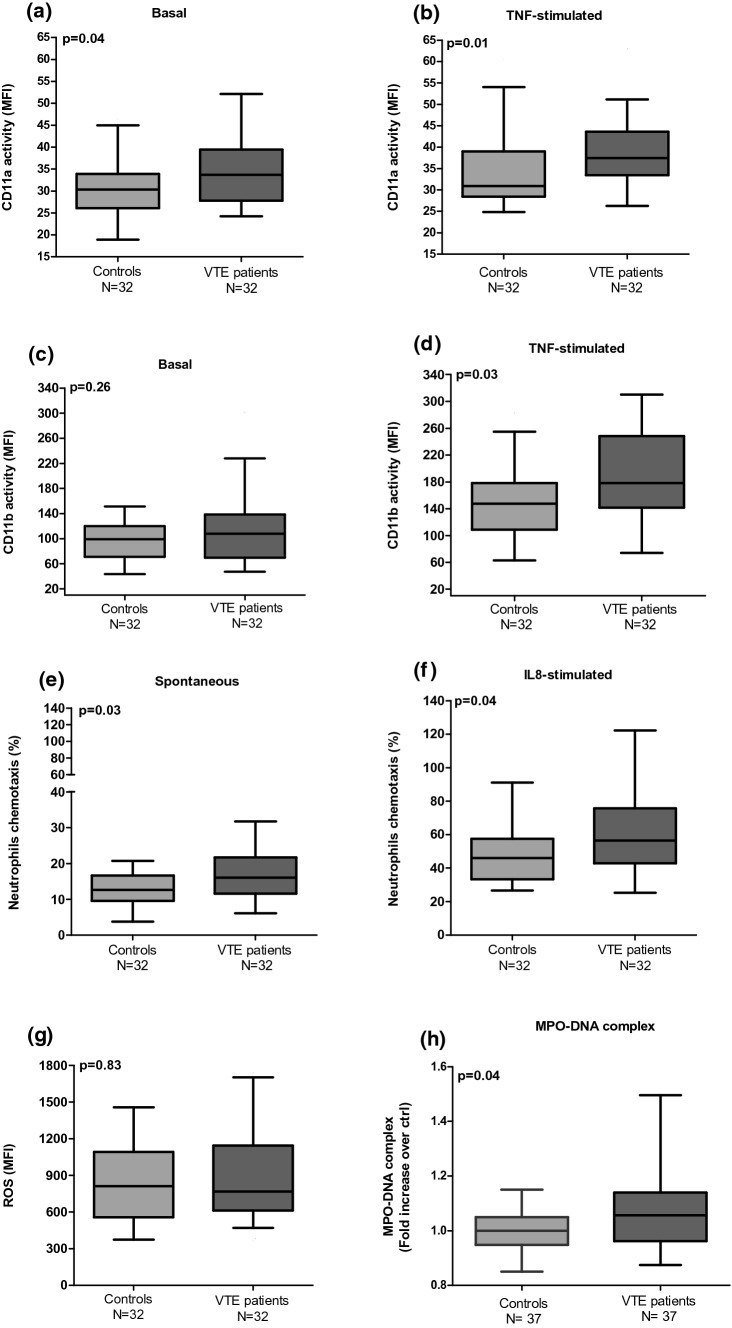
Assessment of neutrophil activation status in VTE patients one year at least after thrombosis compared to controls. **a**, **c** Expression of CD11a (LFA-1) and CD11b (MAC-1) in their activated conformations on basal state and **b**, **d** after stimulation by TNF-a (flow cytometry). **e** Spontaneous and **f** IL-8-stimulated neutrophils chemotaxis. **g** Analysis of neutrophil oxidative stress by ROS production (flow cytometry). **h** NETs activity assessed by DNA complex and neutrophil-derived MPO protein. The p value were calculated by Mann–Whitney U

We also observed a significant increase in the expression of the CD11a subunit, both on basal states (30.30 [IQR 26.05–33.90] vs. 33.70 [IQR 27.83–39.45]) (p = 0.04) and and TNF-α-stimulated states (30.90 [IQR 28.43–39.00] vs. 37.45 [IQR 33.43–43.60]) (p = 0.01) in VTE patients compared to controls (Fig. 2a, b), whereas the CD11b subunit levels increased only after TNFα-inflammatory stimulation (147.59 [IQR 108.90–178.30] × 178.00 [IQR 141.80–248.50]) (p = 0.03) (Fig. 2d).

We further observed that spontaneous and IL-8 stimulated chemotaxis were elevated in the neutrophils from VTE patients when compared to controls (12.65 [IQR 9.62–16.66] vs. 16.07 [IQR 11.62–21.72]); p = 0.03); (46.00 [IQR 33.39–57.53] vs. 56.43 [IQR 42.78–75.64]; p = 0.04, respectively) (Figs. 2e, f).

#### Neutrophil-generated oxidative stress in VTE patients

We investigated whether neutrophil intracellular ROS that is associated with the induction of NETs release [30] was altered in patients with VTE. However, the formation of ROS was similar between patients and controls as shown in Fig. 2g (811.50 [IQR 556.80–1092] vs. 768 [IQR 613–1145]; p = 0.83).

#### NETs activity in VTE patients

The levels of MPO-DNA complexes, indirect biomarkers of NETs release, were significantly increased in VTE patients as compared to controls (1.00 [IQR 0.94–1.05] vs. 1.05 [IQR 0.96–1.13]; p = 0.04) (Fig. 2h).

### ICAM-1 and VCAM-1 soluble adhesion molecules involved in the cross talk between neutrophil and endothelium are increased in VTE patients

**Fig. 3:**
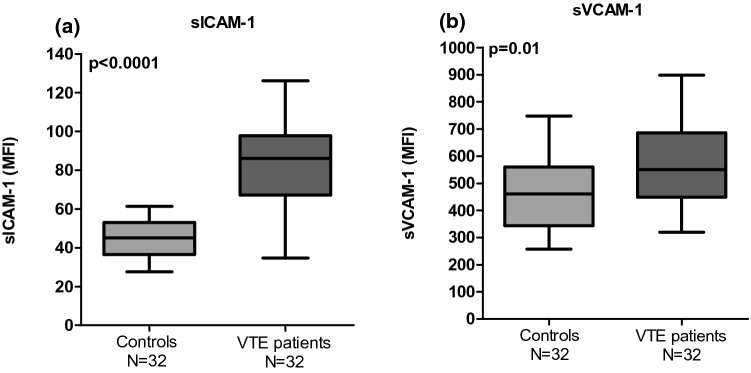
Increased level of soluble endothelial adhesion molecules in serum in VTE patients: **a** sICAM-1 and **b** sVCAM-1. The p value were calculated by Student t and Mann–Whitney U test, respectively

The molecules involved in the cross talk between neutrophils and endothelial cells were evaluated and the results showed a significant increase of sICAM-1 (45.17 [IQR 36.48–52.92] vs. 86.12 [IQR 67.22–97.80]); p < 0.0001) and sVCAM-1 levels (460.20 [IQR 344.10–560.20] vs. 549.50 [IQR 448.70–685.40]); p = 0.01) in patients compared to controls (Fig. 3a, b).

## Discussion

As neutrophil activation and increased NETs were previously demonstrated in the acute phase of VTE [21, 22], in this study they were evaluated at least one year after the clinical event, considering the known long-term complications of VTE. Our results demonstrated that patients with VTE have an increased neutrophil activation characterized by increased expression of the activated epitopes (headpiece of integrin) of the molecules LFA-1 (CD11a) and MAC-1 (CD11b) compared to controls. These patients additionally demonstrated increased spontaneous chemotaxis of neutrophils as well as a more pronounced migratory tendency after inflammatory stimulation by IL-8.

Inflammatory markers, such as TNF-α, IL-6 and IL-8 are increased in patients with prior VTE events [31]. By activating neutrophils-related Rho-GTPase proteins, TNF-α induces the regulation of β_2_ integrin cytoskeletal shape and dynamics, and, subsequently, modulates the activity of this integrin on the neutrophil surface [27].

In a previous study [31], we showed an increase of neutrophil adhesion to fibronectin ligand by static adhesion assay in VTE patients long time after the acute event, but with no increase in the expression of LFA-1 and MAC-1 molecules. Different mechanical strengths and binding avidity to their ligands are observed in the LFA-1 and MAC-1 molecules. The avidity is regulated by integrin affinity and valency of ligand binding [32]. Under normal conditions, these adhesive molecules are found in a low-affinity state for the ligands. However, bidirectional signaling across the cytoplasmatic membrane of cell is responsible for modulation of integrin topological change (headpiece arrangement), as well as, spatial rearrangement and extent over the plasma membrane [33, 34]. Thus, increased integrin affinity corresponds to topological conformational changes in individual integrin heterodimers, which leads to increased ligand-binding energy. While the valency corresponds to the density of integrin heterodimers per area of plasma membrane, this can be dependent on the lateral mobility and cell-surface expression levels [32]. Therefore, the avidity of cell adhesion depends on the equilibrium between affinity state and the valency of a population of adhesive molecules, where the fluctuations or oscillations of individual molecules between low and high-affinity states and valency, probably account for the formation and dissolution of bonds, which is required for migration process complex [35].

Biophysical studies using single-molecule atomic force microscopy have shown that LFA-1/MAC-1 molecules of human and mouse neutrophils present high breaking forces to their respective ligands, indicating long life and high resistance to support the adhesion of neutrophils under physiological flow [36]. However, the binding affinity and mechanical strength for LFA-1/ICAM-1complexes is much higher than that for Mac-1–/ICAM-1 complexes, so that LFA-1/ICAM-1 bonds mainly contribute to maintaining neutrophil adhesion. The LFA-1/ICAM-1 and Mac-1/ICAM-1 interactions mediated cooperatively the neutrophil specific adhesion at rest, but after activation by fMLF, the binding force for high-affinity LFA-1 was sufficient to mediate neutrophil adherence alone and Mac-1 blockade could not reduce adherence. These date indicate that the ability of LFA-1 / Mac-1 to mediate neutrophil adhesion may be directly linked to the mechanical strength (rupture force) and binding kinetics (activation or deactivation rate and affinity) of these receptor-ligand interactions, along with expression and molecular distribution of these molecules. In addition, neutrophil recruitment may also result from tissue- and stimulus-specific interactions between LFA-1 or Mac-1 and their multiple ligands [37].

Therefore, the increased adhesive properties of neutrophils evidenced in our patients may possibly be mediated by enhanced topological affinity of LFA-1 to its ligand, instead of expression level over plasma membrane, and in the presence of TNF-stimulation, both LFA-1 and MAC-1 showed higher activity in these patients than controls. These data are supported by the increase of neutrophil chemotaxis also shown in these patients.

Increased binding activity of LFA-1 and MAC-1 is further related to other post adhesion functions, such as cell spreading, polarization, and intraluminal crawling [38]. Outside-in signaling of Mac-1 ligand bonds are more effective in inducing neutrophil spreading and polarization than LFA-1 ligand bond [37]. Furthermore, before the final step of neutrophil transmigration to inflamed tissues, these cells crawl inside blood vessels seeking preferred sites of transmigration in a MAC-1/ICAM1-bond-dependent manner. When crawling is disabled, transmigration is delayed and occurs preferentially through endothelial-cell junctions (paracellular pathway), mediated by interactions of ICAM-1 and ICAM-2 with LFA1 [39]. These data support our results of LFA-1 increased spontaneous activity (basal) but not of the MAC-1.

Adherent neutrophil can induce the formation of ‘docking structures’ or ‘transmigratory cups’, which are projections of endothelial cells that express high levels of ICAM-1 and VCAM-1[39]. In vitro studies using cultured endothelial cells (HUVECs) showed a significant positive correlation between the soluble forms and the expression of ICAM-1 and VCAM-1 on the surface of these cells, especially under inflammatory conditions, supporting the use of sCAMs as potential biomarkers of endothelial activation and dysfunction [40–43]. Thus, increased levels of sICAM-1 and sVCAM-1 observed in VTE patients suggest not only the presence of exacerbated endothelial activation and dysfunction but also the interaction of neutrophil adhesive molecules with their endothelial ligands, favoring the process of neutrophil migration.

Neutrophil activation is linked to migration from blood circulation to tissue and can be initiated by adhesion to the endothelium, however their effector functions, such as, degranulation, ROS generation, phagocytosis and NETs, only become available once they come into contact with certain ligands (pro-inflammatory cytokines) that can activate other receptors, completing the second activation way [6, 44].

Therefore, in this study markers of ROS generation and NETs were additionally included to investigate neutrophil effector functions. There is evidence that ROS can be involved in the process of NETs release [30]. Herein, we observed an increase in the soluble NETs remnants in VTE patients but not in the levels of ROS compared to controls. Recently, the role of oxidative stress in the formation of NET during venous thrombosis was investigated in Sirt3-/- mice subjected to IVC stenosis. Sirt3 is a deacetylase protein localized mitochondrion, responsible for intracellular regulation of ROS. The authors observed that increased ROS levels, both in mitochondria and cytosol in neutrophils of Sirt3-/- mice did not affect NETs release nor thrombus formation [45], which corroborates with our results, suggesting that ROS may not be involved in the release of NET in VTE.

Several serum assays marking cell-free DNA, nucleosomes, and extracellular DNA co-localized with neutrophil-derived proteins, including the MPO-DNA complex, have been used as biomarkers of NETs in several disorders, including VTE [20–22, 29, 46]. However, many studies on this association have focused only on VTE at diagnosis. In one of the studies, an increase in the MPO-DNA complex and NETotic neutrophils evaluated was observed by flow cytometry in basal conditions in VTE patients compared to healthy individuals [22]. Recently, VTE patients in a stable phase (6 months after the acute event) showed increased levels of cell-free DNA, plasma calprotectin and myeloperoxidase. A correlation was also observed between cell-free DNA with calprotectin [47]. Despite the fact that the marker of cell-free DNA can additionally be derived from dead cells other than neutrophils, in these patients the marker was associated with the presence of NETs. In our study, we used the MPO-DNA complex, which is more specific to quantify soluble NETs remnants [48]. Therefore, our results taken together with these data, not only suggest that patients with VTE at diagnosis have an increased NETs activity, but that this activity remains exacerbated even after a long period (≅ 2 years) of the acute phase of disease.

Evidence shows that NETs and their constituents, such as histones, granular proteins and DNA seem to be essential components in driving prothrombotic activities of activated neutrophils. NETs bind to platelets and red blood cells providing a physical scaffold for thrombus growth[15] and appear to directly stimulate the coagulation cascade [11, 49, 50], as well as the activation and recruitment of platelets[10, 11, 51, 52].

In addition, NETs can further induce endothelial cell death [53] with a major role of histones and myeloperoxidase in this process [14, 54]. The ICAM-1 molecule is susceptible to proteolytic cleavage by neutrophil elastase, and consequently, the soluble form of ICAM-1 is released in the circulation [55, 56]. These data could explain the increase in endothelial dysfunction markers sICAM-1 and sVCAM-1, evidenced in VTE patients in this study.

Endothelial cells, in turn, play a role in inflammatory response, through both direct regulation of neutrophil activity by releasing cytokines (e.g.,IL-1β, IL-8, and ROS) that can accelerate NET formation [53] and modulation of other cellular elements, such as platelets. Massive amounts of von Willebrand factor and P-selectin, which are involved in platelet adhesion and neutrophil recruitment, are secreted during endothelial injury [44, 57]. Hence, we can visualize a situation where the activated endothelium induces the release of NETs, followed by endothelial cytotoxicity, which is responsible, for clearance of mediators that promote more NETs, creating a vicious cycle. This process can result in a systemic chronic inflammation [58], evidenced in the patients of this study by increased plasma levels of C‐reactive protein, an important systemic pro-inflammatory marker.

### Study limitations

Some limitations of this study should be discussed. To begin with, a great number of patients could not be included due to the exclusion criteria such as inflammatory processes and chronic diseases, due to cancer and autoimmune diseases, contributing to the limited sample size and possibly to the fact that some results failed to reach statistical significance. Nevertheless, these cautions were particularly important to assure that the findings were not the result of a secondary process. We restricted the population to the most homogeneous population possible to enhance the validity of our findings. In addition, of these 37 patients, only 32 had enough cell numbers (isolated neutrophils) for neutrophil adhesion, chemotaxis, and ROS assays, as well as 32 serum samples available for endothelial activation test due to blood draw difficulties.

The time lapse between the clinical manifestation of the acute VTE event and the current investigation is variable which may have yielded in heterogeneous results.

Although we have excluded patients with acute VTE, we only evaluated the presence of clinical signs and symptoms of VTE, systematic imaging exams were not performed to exclude asymptomatic VTE.

Another issue was that, despite NETs having been discovered over 10 years ago [9] and having since then been addressed and discussed in different clinical disorders, a gold standard marker has not yet been established. Most methods, not applicable for serum or plasma samples, or require isolation and stimulation of neutrophils in vitro, or have been developed for the detection of NETs in paraffin-embedded tissue. These assays do not represent a real in vivo situation, they are precarious in objectivity, and include problems related to the subjective view of the analyzer and quantification of results. Thus, we decided to include a serological test to quantify NETs remnants in our analysis, with the advantage of not requiring immediate processing and of being technically more straightforward. There are several methods to evaluate soluble NETs remnants. However, some assays such as circulating nucleosomes and cell-free DNA are not specific markers of NETs remnants [48]. Therefore, to solve this problem we included a more accurate assay that evaluates neutrophil-derived proteins (MPO) complexed with DNA. When we designed this study, there was no such standardized kit available on the market. However, this method has been accepted as one of the most specific techniques for monitoring NETs [48]. In addition, NETs remnants, but not intact NETs, can be associated with an increase in coagulation [11] and in the risk of thrombosis [59].

To evaluate MPO-DNA complex, we chose to use serum samples over plasma samples as the latter contains chelators which inhibit the activity of the Ca2+—and Mg2+ -dependent endonucleases. Subsequently, the cleavage of chromatin into oligo- and mononucleosomes (most of the circulating DNA), and the disclosure of antibody binding sites to DNA is impaired, as well as the fact that plasma proteins might react with circulating DNA and mask their presence in plasma. However, higher concentrations de NETs remnants are found in serum compared to plasma [23]. Thus, to avoid these potentially interfering influences, we used serum treated with 10 mM EDTA (pH 8) immediately after centrifugation. EDTA with this concentration seems to inhibit the activity of the DNase I and pH 8 neutralizes the acidification that often accompanies cell death and creates unfavorable conditions for DNase II (activated with pH 4.5) [60]. Therefore, this modification allowed us to work with a more stable serum sample to evaluate the MPO-DNA complex.

Although a significant increase in NETs remnants was observed in patients with a prior VTE, this study was not powered to evaluate whether this difference is clinically relevant.

Finally, we observed a higher WBC count in VTE patients as compared to controls. However, despite being higher among patients, WBC counts were within the normal range (female: 3.9–11.1 × 10^3^/μl/male: 3.7–9.5 × 10^3^/μl). This difference in WBC counts may not have affected our results as the initial concentration of neutrophils was standardized in all assays in both patients and controls.

## Conclusion

In conclusion, to our knowledge, this study demonstrated for the first time that VTE patients, presented increased neutrophil activation patterns even a long period after an acute event, characterized by enhanced activation epitope expression of LFA-1 and MAC-1, chemotaxis and NETs remnants. Increased serum ICAM-1 and VCAM-1 suggested increased cross talk between neutrophil and endothelial cell, as well as, endothelial dysfunction.

## References

[1] Galanaud JP, Monreal M, Kahn SR (2018). Epidemiology of the post-thrombotic syndrome. Thromb Res.

[2] Hansson PO, Welin L, Tibblin G, Eriksson H (1997). Deep vein thrombosis and pulmonary embolism in the general population: ‘the study of men born in 1913’. Arch Intern Med.

[3] Khan F, Rahman A, Carrier M, Kearon C, Weitz JI, Schulman S (2019). Long term risk of symptomatic recurrent venous thromboembolism after discontinuation of anticoagulant treatment for first unprovoked venous thromboembolism event: systematic review and meta-analysis. BMJ.

[4] Hansson PO, Sorbo J, Eriksson H (2000). Recurrent venous thromboembolism after deep vein thrombosis: incidence and risk factors. Arch Intern Med.

[5] Laridan E, Martinod K, De Meyer SF (2019). Neutrophil extracellular traps in arterial and venous thrombosis. Semin Thromb Hemost.

[6] Kazzaz NM, Sule G, Knight JS (2016). Intercellular Interactions as regulators of NETosis. Front Immunol.

[7] Chigaev A, Sklar LA (2012). Aspects of VLA-4 and LFA-1 regulation that may contribute to rolling and firm adhesion. Front Immunol.

[8] Bednarczyk M, Stege H, Grabbe S, Bros M (2020). β2 integrins-multi-functional leukocyte receptors in health and disease. Int J Mol Sci.

[9] Brinkmann V, Reichard U, Goosmann C, Fauler B, Uhlemann Y, Weiss DS (2004). Neutrophil extracellular traps kill bacteria. Science.

[10] Fuchs TA, Abed U, Goosmann C, Hurwitz R, Schulze I, Wahn V (2007). Novel cell death program leads to neutrophil extracellular traps. J Cell Biol.

[11] Engelmann B, Massberg S (2013). Thrombosis as an intravascular effector of innate immunity. Nat Rev Immunol.

[12] Xu J, Zhang X, Pelayo R, Monestier M, Ammollo CT, Semeraro F (2009). Extracellular histones are major mediators of death in sepsis. Nat Med.

[13] Döring Y, Weber C, Soehnlein O (2013). Footprints of neutrophil extracellular traps as predictors of cardiovascular risk. Arterioscler Thromb Vasc Biol.

[14] Qi H, Yang S, Zhang L (2017). Neutrophil extracellular traps and endothelial dysfunction in atherosclerosis and thrombosis. Front Immunol.

[15] Fuchs TA, Brill A, Duerschmied D, Schatzberg D, Monestier M, Myers DD (2010). Extracellular DNA traps promote thrombosis. Proc Natl Acad Sci USA.

[16] Massberg S, Grahl L, von Bruehl ML, Manukyan D, Pfeiler S, Goosmann C (2010). Reciprocal coupling of coagulation and innate immunity via neutrophil serine proteases. Nat Med.

[17] Fuchs TA, Brill A, Wagner DD (2012). Neutrophil extracellular trap (NET) impact on deep vein thrombosis. Arterioscler Thromb Vasc Biol.

[18] Vazquez-Garza E, Jerjes-Sanchez C, Navarrete A, Joya-Harrison J, Rodriguez D (2017). Venous thromboembolism: thrombosis, inflammation, and immunothrombosis for clinicians. J Thromb Thrombolysis.

[19] von Bruhl ML, Stark K, Steinhart A, Chandraratne S, Konrad I, Lorenz M (2012). Monocytes, neutrophils, and platelets cooperate to initiate and propagate venous thrombosis in mice in vivo. J Exp Med.

[20] van Montfoort ML, Stephan F, Lauw MN, Hutten BA, Van Mierlo GJ, Solati S (2013). Circulating nucleosomes and neutrophil activation as risk factors for deep vein thrombosis. Arterioscler Thromb Vasc Biol.

[21] Diaz JA, Fuchs TA, Jackson TO, Kremer Hovinga JA, Lammle B, Henke PK (2013). Plasma DNA is elevated in patients with deep vein thrombosis. J Vasc Surg Venous Lymphat Disord.

[22] Lee KH, Cavanaugh L, Leung H, Yan F, Ahmadi Z, Chong BH (2018). Quantification of NETs-associated markers by flow cytometry and serum assays in patients with thrombosis and sepsis. Int J Lab Hematol.

[23] Holdenrieder S, Stieber P, Bodenmuller H, Fertig G, Furst H, Schmeller N (2001). Nucleosomes in serum as a marker for cell death. Clin Chem Lab Med.

[24] Krabbe J, Beilmann V, Alamzad-Krabbe H, Böll S, Seifert A, Ruske N (2020). Blood collection technique, anticoagulants and storing temperature have minor effects on the isolation of polymorphonuclear neutrophils. Sci Rep.

[25] Canalli AA, Proença RF, Franco-Penteado CF, Traina F, Sakamoto TM, Saad STO (2011). Participation of Mac-1, LFA-1 and VLA-4 integrins in the in vitro adhesion of sickle cell disease neutrophils to endothelial layers, and reversal of adhesion by simvastatin. Haematologica.

[26] Antoniellis Silveira AA, Dominical VM, Morelli Vital D, Alves Ferreira W, Trindade Maranhao Costa F, Werneck CC (2018). Attenuation of TNF-induced neutrophil adhesion by simvastatin is associated with the inhibition of Rho-GTPase activity, p50 activity and morphological changes. Int Immunopharmacol..

[27] Silveira AA, Dominical VM, Lazarini M, Costa FF, Conran N (2013). Simvastatin abrogates inflamed neutrophil adhesive properties, in association with the inhibition of Mac-1 integrin expression and modulation of Rho kinase activity. Inflamm Res.

[28] Bradley PP, Priebat DA, Christensen RD, Rothstein G (1982). Measurement of cutaneous inflammation: estimation of neutrophil content with an enzyme marker. J Investig Dermatol.

[29] Kessenbrock K, Krumbholz M, Schonermarck U, Back W, Gross WL, Werb Z (2009). Netting neutrophils in autoimmune small-vessel vasculitis. Nat Med.

[30] Brinkmann V, Zychlinsky A (2012). Neutrophil extracellular traps: is immunity the second function of chromatin?. J Cell Biol.

[31] Zapponi KC, Mazetto BM, Bittar LF, Barnabe A, Santiago-Bassora FD, De Paula EV (2014). Increased adhesive properties of neutrophils and inflammatory markers in venous thromboembolism patients with residual vein occlusion and high D-dimer levels. Thromb Res.

[32] Ley K, Laudanna C, Cybulsky MI, Nourshargh S (2007). Getting to the site of inflammation: the leukocyte adhesion cascade updated. Nat Rev Immunol.

[33] Hu P, Luo BH (2013). Integrin bi-directional signaling across the plasma membrane. J Cell Physiol.

[34] Luo BH, Carman CV, Springer TA (2007). Structural basis of integrin regulation and signaling. Annu Rev Immunol.

[35] Montresor A, Toffali L, Constantin G, Laudanna C (2012). Chemokines and the signaling modules regulating integrin affinity. Front Immunol.

[36] Li N, Mao D, Lü S, Tong C, Zhang Y, Long M (2013). Distinct binding affinities of Mac-1 and LFA-1 in neutrophil activation. J Immunol.

[37] Li N, Yang H, Wang M, Lü S, Zhang Y, Long M (2018). Ligand-specific binding forces of LFA-1 and Mac-1 in neutrophil adhesion and crawling. Mol Biol Cell.

[38] Pick R, Brechtefeld D, Walzog B (2013). Intraluminal crawling versus interstitial neutrophil migration during inflammation. Mol Immunol.

[39] Phillipson M, Heit B, Colarusso P, Liu L, Ballantyne CM, Kubes P (2006). Intraluminal crawling of neutrophils to emigration sites: a molecularly distinct process from adhesion in the recruitment cascade. J Exp Med.

[40] Pigott R, Dillon LP, Hemingway IH, Gearing AJ (1992). Soluble forms of E-selectin, ICAM-1 and VCAM-1 are present in the supernatants of cytokine activated cultured endothelial cells. Biochem Biophys Res Commun.

[41] Shbaklo H, Holcroft CA, Kahn SR (2009). Levels of inflammatory markers and the development of the post-thrombotic syndrome. Thromb Haemost.

[42] Kjaergaard AG, Dige A, Krog J, Tonnesen E, Wogensen L (2013). Soluble adhesion molecules correlate with surface expression in an in vitro model of endothelial activation. Basic Clin Pharmacol Toxicol.

[43] Witkowska AM, Borawska MH (2004). Soluble intercellular adhesion molecule-1 (sICAM-1): an overview. Eur Cytokine Netw.

[44] Etulain J, Martinod K, Wong SL, Cifuni SM, Schattner M, Wagner DD (2015). P-selectin promotes neutrophil extracellular trap formation in mice. Blood.

[45] Hayashi H, Cherpokova D, Martinod K, Witsch T, Wong SL, Gallant M (2017). Sirt3 deficiency does not affect venous thrombosis or NETosis despite mild elevation of intracellular ROS in platelets and neutrophils in mice. PLoS ONE.

[46] Miyoshi A, Yamada M, Shida H, Nakazawa D, Kusunoki Y, Nakamura A (2016). Circulating neutrophil extracellular trap levels in well-controlled type 2 diabetes and pathway involved in their formation induced by high-dose glucose. Pathobiology.

[47] Martos L, Oto J, Fernández-Pardo Á, Plana E, Solmoirago MJ, Cana F (2020). Increase of neutrophil activation markers in venous thrombosis-contribution of circulating activated protein C. Int J Mol Sci.

[48] Masuda S, Nakazawa D, Shida H, Miyoshi A, Kusunoki Y, Tomaru U (2016). NETosis markers: quest for specific, objective, and quantitative markers. Clin Chim Acta.

[49] Noubouossie DF, Whelihan MF, Yu YB, Sparkenbaugh E, Pawlinski R, Monroe DM (2017). In vitro activation of coagulation by human neutrophil DNA and histone proteins but not neutrophil extracellular traps. Blood.

[50] Stavrou EX, Fang C, Bane KL, Long AT, Naudin C, Kucukal E (2018). Factor XII and uPAR upregulate neutrophil functions to influence wound healing. J Clin Investig.

[51] Xu J, Zhang X, Monestier M, Esmon NL, Esmon CT (2011). Extracellular histones are mediators of death through TLR2 and TLR4 in mouse fatal liver injury. J Immunol.

[52] Fuchs TA, Bhandari AA, Wagner DD (2011). Histones induce rapid and profound thrombocytopenia in mice. Blood.

[53] Gupta AK, Joshi MB, Philippova M, Erne P, Hasler P, Hahn S (2010). Activated endothelial cells induce neutrophil extracellular traps and are susceptible to NETosis-mediated cell death. FEBS Lett.

[54] Saffarzadeh M, Juenemann C, Queisser MA, Lochnit G, Barreto G, Galuska SP (2012). Neutrophil extracellular traps directly induce epithelial and endothelial cell death: a predominant role of histones. PLoS ONE.

[55] Champagne B, Tremblay P, Cantin A, St PY (1998). Proteolytic cleavage of ICAM-1 by human neutrophil elastase. J Immunol.

[56] Robledo O, Papaioannou A, Ochietti B, Beauchemin C, Legault D, Cantin A (2003). ICAM-1 isoforms: specific activity and sensitivity to cleavage by leukocyte elastase and cathepsin G. Eur J Immunol.

[57] Michels A, Albánez S, Mewburn J, Nesbitt K, Gould TJ, Liaw PC (2016). Histones link inflammation and thrombosis through the induction of Weibel-Palade body exocytosis. J Thromb Haemost.

[58] Dzikowska-Diduch O, Domienik-Karłowicz J, Górska E, Demkow U, Pruszczyk P, Kostrubiec M (2017). E-selectin and sICAM-1, biomarkers of endothelial function, predict recurrence of venous thromboembolism. Thromb Res.

[59] Jiménez-Alcázar M, Limacher A, Panda R, Méan M, Bitterling J, Peine S (2018). Circulating extracellular DNA is an independent predictor of mortality in elderly patients with venous thromboembolism. PLoS ONE.

[60] Barry MA, Eastman A (1993). Identification of deoxyribonuclease II as an endonuclease involved in apoptosis. Arch Biochem Biophys.

